# A Cross-Sectional Study on the Impact of Dental Fear and Anxiety on the Quality of Life of Romanian Dental Students

**DOI:** 10.3390/medicina61040688

**Published:** 2025-04-09

**Authors:** Adina Oana Armencia, Irina Bamboi, Bianca Toader, Anca Rapis, Andrei Nicolau, Carina Balcos, Walid Edlibi Al Hage, Tinela Panaite

**Affiliations:** 1 Department of Surgery, Faculty of Dental Medicine, “Grigore T. Popa” University of Medicine and Pharmacy, 700115 Iasi, Romania; adina.armencia@umfiasi.ro (A.O.A.); irina.bamboi@umfiasi.ro (I.B.); bianca_toader@umfiasi.ro (B.T.); carina.balcos@umfiasi.ro (C.B.); tinela-panaite@umfiasi.ro (T.P.); 2 Department of Implantology, Removable Prosthesis, Denture Technology, Faculty of Dental Medicine, “Grigore T. Popa” University of Medicine and Pharmacy, 700115 Iasi, Romania; anca.stupu@umfiasi.ro (A.R.); edlibi.walid@umfiasi.ro (W.E.A.H.)

**Keywords:** IDAF questionnaire, DAS-R CORAH questionnaire, WHOQOL-BREF questionnaire

## Abstract

*Background and Objectives*: Dental anxiety influences quality of life, causing emotional and physical discomfort. The aim of this study was to identify the prevalence of dental anxiety among young adults and how it influences the dimensions of quality of life, especially in terms of physical and psychological health. *Material and Methods*: This study was conducted between March and July 2024, within the Community Dentistry Discipline at the “Grigore T. Popa” University of Medicine and Pharmacy in Iași, on a group of 180 students to assess dental anxiety and its impact on their quality of life. Participants completed questionnaires using scales such as the IDAF, DAS-R CORAH, and WHOQOL-BREF. *Results:* Most participants presented a moderate level of dental anxiety, with 59.4% without anxiety and 6.1% with severe anxiety. Significant correlations were established between dental anxiety and physical and psychological dimensions of quality of life. In contrast, there were no significant correlations between dental anxiety and social relationships or the environment. Anxiety and fear were negatively correlated with perceived physical well-being, but positive correlations were also identified between sensitivity to dental stimuli and more favorable perceptions of physical and psychological health. Discussion: Dental anxiety has a significant impact on patients’ physical and psychological health, especially on their perception of their well-being. This study confirms previous research showing that dental anxiety is related to certain dental procedures. *Conclusions*: Dental anxiety has a significant impact on psychological and physical perception but does not significantly affect social interactions.

## 1. Introduction

Although dentistry has evolved a lot in recent years, there are still many people who believe that dental treatment causes emotions or pain. These perceptions are the main causes of anxiety and fear in many patients [1].

While dental phobia, also known as a fear of the dentist, is an irrational fear that prevents patients from engaging in situations that cause intense fear (in this case, situations related to dental care and dental treatments), dental anxiety is defined as a patient’s response to dental conditions that cause stress [2,3].

Fear is a normal and adaptive emotional reaction to an identifiable threat (e.g., the anticipation of pain or dental treatment). People who experience dental fear have poorer oral health than those who do not [4], postponing long-term dental treatments and postponing dental visits [5], leading to oral pain or even the need for invasive and potentially painful procedures. This behavior can also lead to a vicious cycle of dental fear: postponing visits causes the condition to continue to progress, which subsequently leads to emergency treatments, which in turn exacerbate or maintain the person’s dental fear [6].

Dental anxiety is a generalized feeling of uneasiness or stress related to visits to the dentist, without a clear or immediate cause [7].

Dental phobia is a severe psychological disorder, recognized (e.g., in the DSM-5) as a specific phobia. Dental phobia is the fear associated with therapeutic procedures, medical personnel, or even the environment in the dental office, affecting oral health behaviors [8]. It is one of the main causes of treatment avoidance, which can lead to poor oral health. Generically, a phobia is a persistent, intense, and exaggerated fear of a specific stimulus, leading to the avoidance of the perceived danger. In the International Statistical Classification of Diseases and Related Health Problems (ICD)-10, dental phobia is considered an overwhelming and irrational fear of dentistry [9].

Various dental treatments can induce physiological and behavioral changes, with a higher degree of anxiety frequently being associated with interventions such as cavity preparation with high-speed rotary instruments, the use of ultrasonic scaling or root canal treatments, and especially tooth extraction [10].

Daily oral hygiene is essential for maintaining dental health, preventing dental diseases and improving quality of life, but it also influences the degree of integration and social acceptance. However, compliance with these practices is often difficult when dental anxiety is present. This creates a vicious circle that associates feelings of shame with existing oral problems and with the continued avoidance of necessary treatments [11,12,13]. In addition, research by Gatchell and his collaborators showed that 70% of patients who come to the dental office have feelings of fear, while 15% avoid regular dental check-ups due to fear and anxiety [14].

All types of fear are characterized by four elements: the fear of physical harm, fear of the unknown, fear of losing control, and fear of dependence and helplessness [15]. In addition, the pain tolerance threshold varies from one person to another, being influenced, among other things, by the emotional state of the person in question [16,17]. Accurately identifying the causes that produce fear, anxiety, or dental phobia is essential to create more effective treatment for these patients.

It is also essential to understand in detail the elements that contribute to these states of fear. Previous experiences, rumors, or other societal influences can cause them [1,18]. Also, the patient’s socio-economic status and level of education have a significant impact on their degree of dental fear and anxiety, and there are variations depending on these [19].

Therefore, to effectively manage patient anxiety, it is essential that a trusting relationship is established between the patient and the doctor, allowing for honest and ongoing communication. Proper communication can significantly reduce anxiety levels, while its absence can have the opposite effect [19]. The patient’s first contact is with the medical team, who must pay attention to both emotional and objective aspects. By analyzing the patient’s rhythm, tone, volume, and speed of speech, staff can obtain essential information. Dentists can reassure anxious patients through demonstrating empathy, active listening, and clarifying procedures in simple and calming language. These approaches help to ‘normalize’ the patient’s fear, fostering a more reassuring clinical environment [20].

Age, gender, and education level play an important role in the onset and progression of dental fear and anxiety. The elderly generally have a lower level of anxiety than the young [13]. Women are more likely to experience dental anxiety and phobia [13], although their pain tolerance threshold is increased [21,22]. Men, on the other hand, feel pressure due to socio-cultural norms and may not report signs of fear or anxiety during dental treatments.

Parental behavioral patterns influence children’s feelings of fear and dental anxiety. It is believed that one of the main sources that contribute to the development of dental fear in children is the “parental pathway” [23,24]. Studies in the literature show that certain types of fears and phobias can, to some extent, be transmitted from the family environment. This affects the neural processes that lead to the development of fear in the brain. While direct conditioning and the emotional state of the child are considered important factors, it has been found that a family member who experiences great dental fear can contribute to the development of dental anxiety or phobia in childhood, being a predictive factor for dental phobia in future adults.

The dental anxiety of parents influences the development and manifestation of dental anxiety in children. A study conducted in six European countries by Simunovic et al. highlighted the link between the level of dental anxiety of parents and that of children. According to the results of the study, parents who show high levels of dental anxiety tend to transmit the same behavior to their children [25], either due to a genetic or environmental predisposition (genetic predisposition, personality traits, age, sex, and gender) or due to education.

Some children may develop anxiety even before they have come into direct contact with negative experiences or information. This may be the result of exposure to negative information, most often from television, parents, family members, teachers, or social media. It should also be remembered that anxiety in children may be the result of observing and imitating the anxious behavior of adults [26].

Additionally, existing studies in the literature have shown that parents who experience dental pain or have a negative perception of dental treatments can transmit these feelings to their children, increasing the risk of developing dental anxiety [27]. Thus, the way parents express their fears and avoid going to a dentist can affect children’s attitudes and behaviors regarding dental care [23].

Dental trauma can have social and emotional implications that influence treatment methods. There are studies in the literature [28,29] that attest the high probability of the psycho-logical impact resulting from traumatic events causing the appearance of dental fear and anxiety, leading to the impairment of individuals’ quality of life. Thus, a study conducted in Croatia in 2022 found that 12.5% of children who suffered various types of dental trauma showed signs of severe anxiety [30]. In a study conducted by Kvesic et al., they highlighted the fact that a percentage of 14.55–17.72% of children with trauma showed dental fear and anxiety [31].

If this is not addressed, individuals’ oral health may worsen, and their quality of life may decrease as a result of untreated oral conditions [7].

Quality of life refers to the degree of satisfaction that a person feels about their life. This concept has often been associated with the general health of the individual [32] and has subsequently been extended to include aspects related to oral health. It is a complex concept that includes a personal assessment of oral health, functional and emotional states, and the expectations of and level of satisfaction with the care received, as well as your self-image [33]. This concept describes how oral health affects a person’s psychological and social state. Consequently, fear causes the avoidance of dental treatments, which leads to a deterioration in oral health, an increase in social embarrassment, fear, and anxiety, which ultimately affects the quality of life. Therefore, quality of life can be improved by the effective treatment of dental fear and anxiety. Better understanding their causes and effective communication with patients, with a focus on emotional and social experiences, can lead to safer and more effective interactions [34,35].

Anxiety and fear are deeply personal feelings that can vary significantly in intensity, severity, and how they manifest from one person to another. An unbiased assessment of these experiences is difficult because of this subjectivity. Furthermore, the diversity of anxiety traits makes them even more difficult to understand and interpret [36].

There are situations where even those who provide medical treatment, such as dental students, can experience dental fear and anxiety. A study conducted among Brazilian dental students revealed that 27.5% of them experienced fear when they became patients. Dental students’ anxiety and fear can negatively affect patients’ attitudes towards dental treatments, leading to the refusal or avoidance of necessary treatments [37]. Women are more frequently affected by dental anxiety than men, although in most cases, the data are not statistically significant. Al-Omari studied students from various fields and found that dental students have lower levels of dental anxiety compared to those from other fields [38].

The aim of this study was to assess the prevalence of dental fear and anxiety among dental students and how these factors influence different areas of quality of life. It was hypothesized that there is a significant correlation between the level of dental anxiety and the quality of life of dental students. We also added an alternative hypothesis, “High levels of dental anxiety are associated with a negative perception of health status among dental students”.

## 2. Materials and Methods

### 2.1. Study Design and Study Group

This cross-sectional descriptive study was conducted between March 2024 and July 2024, within the Community Dentistry Discipline, Faculty of Dental Medicine, University of Iași, Romania, with the approval of the Ethics Committee of the “Grigore T. Popa” University of Iași (No. 393/31 January 2024).

To calculate the sample size, we applied the calculation formula used for a confidence level of *p* = 95% and z = 1.96, with a margin of error of 5% [39], to the total number of students in the 5th year of the Romanian-language teaching series. The result was 100 subjects. Since several students voluntarily expressed their desire to participate in the study, the final sample consisted of 180 students from this academic year.

The inclusion criteria for the study were students aged between 18 and 30 and students willing to complete the questionnaires, as well as the absence of treatments or medications that could influence their quality of life. The exclusion criteria for the study were incomplete questionnaires and specific medical conditions, as well as students with a history of non-compliance.

A clear description of the purpose of the research was given, emphasizing that participation in the study was voluntary and that the information provided by the participants would be treated with discretion and anonymity. An interactive presentation was used to provide additional information about the study to be conducted, combining a face-to-face discussion with a PowerPoint presentation. This provided additional data on both how to complete the questionnaires and how to calculate the final score. Then, the subjects participating in the study signed the informed consent form and then completed the questionnaires.

### 2.2. The Questionnaire

To assess the level of fear and anxiety, as well as how they affect the quality of life, a questionnaire method was used. Specifically, to assess the degree of fear, the Index of Dental Anxiety and Fear (IDAF) and the Depression Anxiety Stress Scales (DASS) were used, and to assess the degree of dental anxiety, the Corah index was used. We used both indicators; the DAS-R measures the intensity of dental anxiety/treatment fear, focusing on the patient’s emotional reaction to common dental procedures, while the Dental Anxiety and Fear Index (IDAF-4C+) analyzes multiple facets of dental anxiety and fear, covering all four components of the fear response: behavioural, cognitive, emotional, and physiological.

Quality of life was assessed using the World Health Organization Quality of Life, BREF (WHOQOL-BREF), indicator.

The Index of Dental Anxiety and Fear (IDAF) comprises three independent modules, including (1) the Core Module (IDAF-4C), which is the main module of the larger measurement; (2) the Phobia Module (IDAF-P), which uses diagnostic criteria based on the DSM-V; and (3) the Stimulus Module (IDAF-S), which requires an assessment of the intensity of anxiety related to various dental stimuli [40,41,42].

The Phobia and Stimulus Modules were designed to be used not as scales, but for epidemiological and clinical purposes. The IDAF-4C Core Module contains eight questions, each with two items addressing the behavioral, emotional, cognitive, and physiological components of dental anxiety and fear. Responses to the IDAF-4C questions range from “I strongly disagree” (1) to “I strongly agree” (5), with higher scores indicating greater dental fear. The mean scores of the full scale were categorized as follows: “No or little dental fear” (score range of 1–1.5), “Low dental fear” (score range of 1.51–2.5), “Moderate dental fear” (score range of 2.51–3.5), and “High dental fear” (score > 3.5) [40,41,42].

The Depression Anxiety Stress Scales (DASS) is a widely used questionnaire for dental anxiety [43]. It contains four questions: the first two questions measure anxiety the day before treatment or just before the start of treatment, and the next two questions measure anxiety during two common dental treatments. There are five response options for each question, coded from 1 to 5, and the DAS score represents the sum of the responses. Thus, the level of dental anxiety is low for values less than 9, moderate for values between 9 and 12, high for values between 13 and 14, and extremely high for values above 15 [44].

The WHOQOL-BREF (World Health Organization Quality of Life—BREF) is a tool developed by the World Health Organization (WHO) to assess the quality of life [45,46]. It includes 26 questions and covers 4 main areas (Table 1):

1. Physical domain: Assesses aspects of physical health, such as energy levels, pain, sleep, mobility, and the ability to perform daily activities.
2. Psychological domain: Measures emotional states, self-image, positive and negative feelings, the ability to concentrate, and personal satisfaction.
3. Social relationships domain: Focuses on interpersonal relationships, social support, and satisfaction with an individual’s social life and the support provided by family and friends.
4. Environmental domain: Includes safety, housing, financial resources, access to medical care, recreational opportunities, and job satisfaction.

The scores calculated for each domain (and implicitly for the general quality of life) are obtained by summing the responses to the corresponding questions and subsequently converting them into standardized scores, according to the instructions provided by the WHO [47].

Each raw scale score is then converted to a score on a scale from 0 to 100 using the following formula [48]:

$$Transformed\;scale=\frac{\left(Actual\;raw\;score-lowest\;possible\;raw\;score\right)}{Possible\;raw\;score\;range}\times 100$$

where the “Actual raw score” represents the values obtained through summation, the “lowest possible raw score” is the minimum possible value that could be obtained through summation (this value would be 4 for all facets), and the “Possible raw score range” is the difference between the maximum possible raw score and the minimum possible raw score (this value would be 16 for all facets: 20 minus 4). This results in the transformation of the minimum and maximum possible scores into 0 and 100, respectively, values that represent the percentage of the total possible score obtained.

Testing the normality of the data distribution was performed using the Kolmogorov–Smirnov test (with Lilliefors correction), recommended for groups larger than 100 participants. All questionnaires used were validated for the Romanian population. Thus, the internal consistency of the IDAF indicator was 0.945 [41]. The DAS questionnaire presented an acceptable consistency, having a Cronbach alpha coefficient of 0.78, according to the study conducted by Sfeatcu et al. [49]. Also, the average Cronbach alpha coefficient for the WHOQOL-BREF indicator was 0.92 [46].

### 2.3. Statistical Analysis

Statistical analysis was performed using SPSS 26.0 (IBM, Armonk, NY, USA), using both descriptive and inferential methods, to highlight the relationships between dental anxiety and socio-demographic variables, respectively, and quality of life. Frequency and percentage distributions were used to describe the socio-demographic characteristics of the sample (age, sex, background, socio-economic level, and frequency of dental check-ups). To compare the scores obtained on the IDAF and DAS-R CORAH questionnaires according to the independent variables, an ANOVA test was used. Regarding the analysis of IDAF scores, the means and standard deviations were calculated for each component: the IDAF-C (Core Module), IDAF-P (Phobia and Anxiety Components), and IDAF-S (Stimuli Module), respectively. Correlations between dental anxiety scores (IDAF and DAS-R CORAH) and quality of life scores (WHOQOL-BREF) were analyzed using the Pearson coefficient. The statistical significance level was set at *p* < 0.05.

## 3. Results

### 3.1. The Demographic Characteristics of the Study Group

The study group consisted of 180 participants, the majority belonging to the 19–29 age group (66.7%), while 33.3% belonged to the 30–39 age group. The gender distribution was relatively balanced, with 48.9% men and 51.1% women. In terms of their residence, participants from urban areas predominated (61.7%), compared to 38.3% from rural areas. In terms of their socio-economic level, almost half of the participants had a high status (48.9%), while 47.2% fell into the medium-level category, and a small percentage (3.9%) had a low socio-economic level. Regarding dental visits, 60.6% of participants had had a dental check-up in the last year, while 39.4% had not been to the dentist for over a year, suggesting a moderate frequency of check-ups, but with a significant percentage of people who did not periodically check their oral health (Table 2).

### 3.2. Prevalence of Fear and Anxiety

The analysis of dental anxiety levels according to the DAS-R CORAH scale indicated that 59.4% of participants did not have dental anxiety, while 26.1% had moderate anxiety, 8.3% had high anxiety, and 6.1% had severe anxiety. In terms of gender, women had a higher prevalence of severe dental anxiety (72.7%) compared to men (27.3%), but the differences between genders were not statistically significant (*p* = 0.760, r = −0.072, F = 0.384), suggesting a weak association between gender and dental anxiety (Table 3).

In terms of socio-economic status, people with a high socio-economic status predominated in the categories of no anxiety (50.5%) and moderate anxiety (46.8%), while people from the middle class had a higher frequency of severe anxiety (54.5%). However, the values of *p* = 0.800, r = 0.061, and F = 0.632 indicate a very weak association between socio-economic status and dental anxiety.

Regarding the frequency of dental check-ups, it was observed that people who had been to the dentist in the last 12 months had a higher prevalence of mild anxiety (66.4% of those without anxiety and 44.7% of those with moderate anxiety), while those who had not been to the dentist for over a year tended to show a higher level of severe anxiety (55.3% of those with moderate anxiety and 40.0% of those with high anxiety). However, the *p* value = 0.067 and the coefficient r = 0.047 suggest that the time elapsed since the last dental check-up was not a significant predictor of dental anxiety. In conclusion, although there were descriptive differences between groups, no statistically significant relationships were identified between dental anxiety and the variables analyzed, such as gender, socio-economic status, and the time elapsed since the last dental visit.

The statistical analysis of the mean scores and standard deviations for the variables assessed in the IDAF and specific dental anxiety factors highlighted the main concerns of the participants (Table 4).

The IDAF scores indicate a moderate level of dental anxiety, with a mean score of 1.9050 (SD = 1.07950) for the IDAF-C (Core Module), suggesting quite high variability between participants. In the IDAF-P (Phobia Module—Phobia Component) and IDAF-P (Phobia Module—Anxiety Component), the mean scores were 1.7444 (SD = 0.43739) and 1.8833 (SD = 0.32192), indicating a relatively constant level of phobias and anxiety related to dental treatments. The Stimuli Module (IDAF-S) had a mean score of 2.5261 (SD = 0.86266), indicating that patients perceived external stimuli as moderately anxiety-provoking.

Among the specific anxiety factors, the cost of treatment (mean = 3.2333, SD = 1.25560) was the main concern, suggesting that the financial aspect is a significant cause of stress for patients. Other notable sources of anxiety were gagging/choking (mean = 2.8944, SD = 1.42796), pain (mean = 2.7444, SD = 1.12425), and feeling sick or disgusted (mean = 2.7056, SD = 1.33996), suggesting that patients are more susceptible to physical discomfort and involuntary reflexes during dental procedures.

Other important factors included a doctor lacking empathy (mean = 2.6833, SD = 1.30951) and a fear of injections (mean = 2.3778, SD = 1.35842). On the other hand, anxieties related to feeling out of control (mean = 2.2333, SD = 1.09901), uncertainty about treatment (mean = 2.1889, SD = 1.27191), and feeling numb (mean = 2.0778, SD = 1.27483) were less intense, but still relevant.

**Figure 1:**
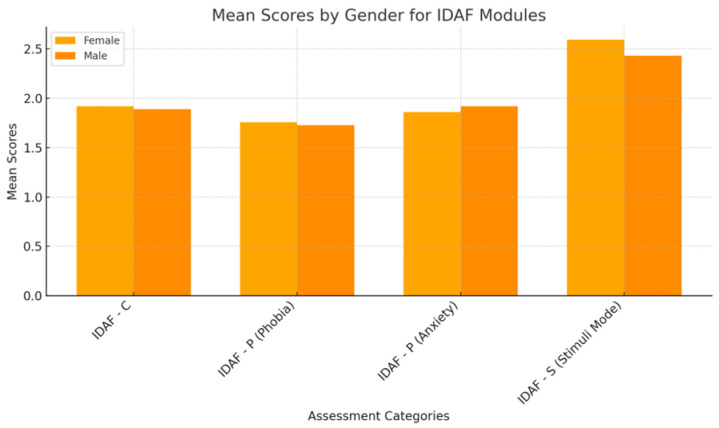
IDAF mean scores by gender.

The analysis of the IDAF (Index of Dental Anxiety and Fear) scores according to individuals’ gender, socio-economic level, and time since their last dental visit did not reveal statistically significant differences between groups (Figure 1).

Regarding gender, women had slightly higher scores on the IDAF-C (Core Module score, M = 1.9168) and IDAF-S (Stimuli Module, M = 2.5925) compared to men (IDAF-C: M = 1.8877, IDAF-S: M = 2.4288). Also, on the IDAF-P (Phobia Module—Anxiety Component), men had a slightly higher mean score (1.9178 compared to 1.8598 in women), but the differences were not statistically significant (*p* > 0.05), which suggests that gender does not significantly influence dental anxiety.

**Figure 2:**
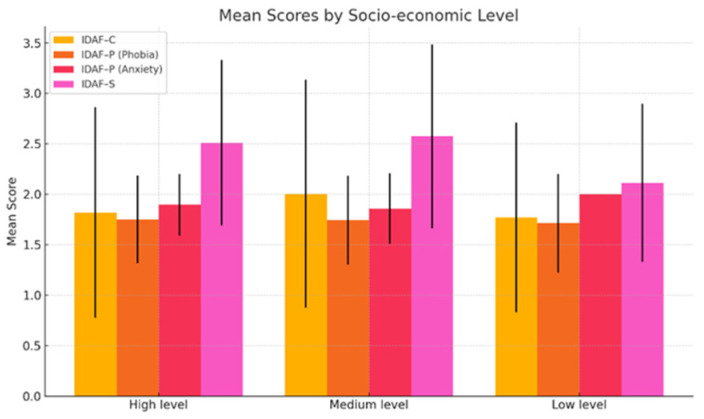
IDAF mean scores by socio-economic level.

Depending on their socio-economic level, participants in the middle-level category had higher scores on the IDAF-C (2.0035) and IDAF-S (2.5765) compared to those with high (1.8206 and 2.5102) and low (1.7700 and 2.1143) socio-economic levels. However, the *p* values for all dimensions (*p* > 0.05) indicated a statistically insignificant relationship between dental anxiety and socio-economic status (Figure 2).

**Figure 3:**
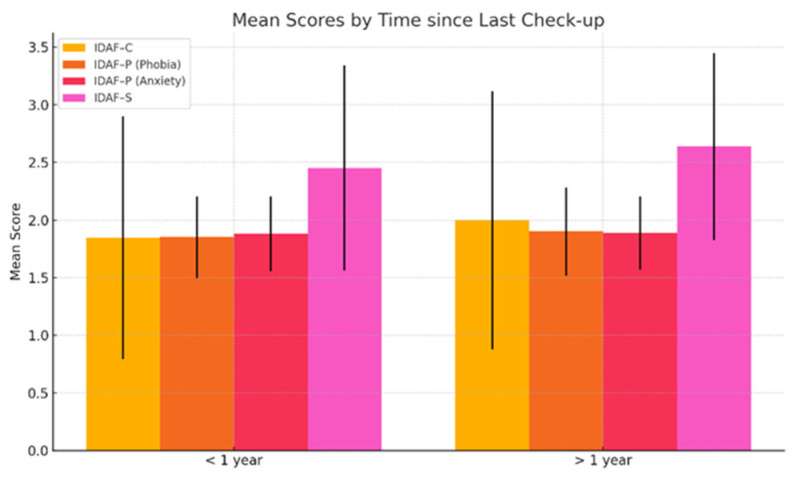
IDAF mean scores by last dental check-up.

When analyzing the time elapsed since the last dental visit, people who had not been to the dentist in the last year tended to have slightly higher scores on all IDAF dimensions (e.g., IDAF—C: 1.997 vs. 1.845; IDAF—S: 2.639 vs. 2.452), suggesting slightly increased dental anxiety among those who avoid regular dental visits. However, *p* values > 0.05 indicated that the differences were not statistically significant, and the time elapsed since the last dental check-up cannot be considered a clear predictor of dental anxiety (Figure 3).

### 3.3. Quality of Life Analysis

The analysis of the quality of life allowed us to obtain, for the “Physical Health” domain, a score of 44.04, which suggests a moderate state of physical health. The score of 63.95 obtained for the “Psychological” domain was relatively higher compared to that for physical health, which highlights a fairly good psychological state, emotionally balanced, with a feeling of general satisfaction. The score of 41.25 for the “Social Relationships” domain shows a moderate social life, in which interaction with those around you can improve, either by deepening existing relationships or by developing new connections. A high score was obtained in the “Environment” domain, which indicates a very high satisfaction with the environment in which the subjects carry out their daily activities, being considered an important factor in their general well-being (Table 5).

The total score (74.4) suggests a fairly good overall quality of life, but with room for improvement, especially in the areas of physical health and social relationships.

### 3.4. Dental Anxiety–Quality of Life Correlation

The analysis of correlations between WHOQOL-BREF domains and DAS-R CORAH and IDAF scores highlighted significant relationships between dental anxiety and certain dimensions of quality of life (Table 6).

Regarding the physical domain, a weak negative correlation was observed between DAS-R CORAH and IDAF-C scores (−0.136, *p* = 0.068), suggesting that individuals with higher dental anxiety may have a slightly more negative perception of their physical condition, although the relationship was not statistically significant. However, IDAF-S (Stimuli Module) scores showed a significant positive correlation with the physical domain (0.184, *p* = 0.014), indicating that individuals more sensitive to dental stimuli may have a more favorable perception of their physical health.

For the psychological domain, significant correlations were identified. DAS-R CORAH scores were positively correlated with the psychological domain (0.198, *p* = 0.008), suggesting that a higher level of dental anxiety is associated with a more positive perception of an individual’s psychological well-being. This correlation was, however, weak, indicating that higher DAS-R scores (reflecting greater symptom severity) were only slightly associated with corresponding changes in the psychological quality of life. Because the strength of the relationship was low, this result suggests that the psychological domain of quality of life may be influenced by factors other than those assessed by the DAS-R scale (e.g., social support, self-esteem, individual coping strategies, socio-economic status, or general health).

In contrast, IDAF-P (Phobia Module—Anxiety Component) scores presented a moderate negative correlation (−0.241, *p* = 0.001), indicating that individuals with a high level of phobic anxiety perceive a more negative psychological state. IDAF-S scores were also positively correlated with this domain (0.221, *p* = 0.003), suggesting that sensitivity to dental stimuli is associated with a more favorable psychological perception.

The analysis for the social relations domain did not indicate significant correlations, with weak and statistically insignificant coefficients (*p* > 0.05). For example, the correlation between DAS-R CORAH scores and this domain was negative (−0.135, *p* = 0.071), indicating an insignificant association between dental anxiety and the perception of social relations (Table 6).

For the environmental domain, a significant positive correlation was identified between IDAF scores and this domain (0.198, *p* = 0.008), indicating that individuals more sensitive to dental stimuli may have a more favorable perception of environmental factors. In contrast, DAS-R CORAH and IDAF-C scores did not have significant correlations with this domain.

The results of this study support the hypothesis that there is a significant correlation between dental fear and anxiety and quality of life, as statistically significant correlations were identified, especially in the psychological and physical domains of quality of life. DAS-R CORAH scores were positively correlated with the psychological domain (*p* = 0.008), and IDAF-P (Anxiety Component) scores had a moderate negative correlation with this domain (*p* = 0.001), suggesting that dental anxiety influences the perception of psychological states. In addition, the IDAF-S scores showed a significant positive correlation with the physical domain (*p* = 0.014), indicating that people who are more sensitive to dental stimuli perceive their physical health differently. On the other hand, the social relations domain did not show significant correlations, suggesting that dental anxiety does not strongly affect social interactions. Therefore, although the impact of dental anxiety on quality of life was not uniform, the existence of significant relationships requires the reconsideration of the initial hypothesis and reformulation of the conclusions to reflect these partial correlations.

If we summarize the results obtained, we can say that in the physical domain, DAS-R scores showed a weak negative correlation with the physical health perception (r = −0.136, *p* = 0.068), suggesting a slight tendency for dental anxiety to be associated with a less favorable perception of physical health. In contrast, IDAF-S (Stimuli Module) scores showed a significant positive correlation with this domain (r = 0.184, *p* = 0.014), indicating that individuals more sensitive to dental stimuli may perceive more favorable physical health. In the psychological domain, both anxiety measurement instruments had significant correlations, but with opposite directions: the DAS-R CORAH was positively correlated with psychological states (r = 0.198, *p* = 0.008), suggesting that higher dental anxiety is associated with a better perception of psychological well-being, while the IDAF-P (Anxiety Component) showed a moderate negative correlation (r = −0.241, *p* = 0.001), indicating that severe anxiety is associated with a more negative perception of psychological states. In the social relations domain, neither the DAS-R nor IDAF showed significant correlations, suggesting that dental anxiety did not significantly influence the participants’ social interactions. In the environmental domain, the IDAF-S showed a significant positive correlation (r = 0.198, *p* = 0.008), suggesting that sensitivity to dental stimuli may be associated with a more favorable perception of the environment, while the DAS-R did not have a significant correlation. These results indicate that dental anxiety significantly influences the psychological and physical domains of quality of life, and sensitivity to dental stimuli may have an important role in the perception of well-being of individuals.

## 4. Discussion

The analysis of the level of dental anxiety according to the DAS-R CORAH scale showed that approximately half of the study participants did not have dental anxiety. Female subjects had a higher prevalence of severe dental anxiety. Also, people with a high socio-economic status did not have dental anxiety, and those who had been to the dentist in the last 12 months had a higher prevalence of mild anxiety.

Studies have shown a higher prevalence of dental anxiety in female subjects [50,51], possibly because this type of anxiety in women can be associated with other phobias or generalized anxiety. Alansaari obtained similar results in a study conducted in the United Arab Emirates [52].

Age plays an important role in the occurrence of dental anxiety. Thus, young subjects usually present higher dental anxiety compared to older people (probably due to the greater number of experiences at the dentist) [53,54].

In addition, people with a high level of education experience less fear of dental treatments, as they have a higher level of understanding of therapeutic procedures compared to people with a lower level of education [54]. Studies conducted by Alansari [2023] in the United Arab Emirates and Muneer [52] in Pakistan showed that patients who had engaged in academic studies presented higher levels of anxiety. On the contrary, Peric and Tadic found a significantly increased level of dental anxiety in participants with a lower level of education [55].

Numerous previous studies have suggested that individuals with a lower socio-economic status exhibit higher levels of anxiety, highlighting the influence of this factor on dental anxiety [56,57].

A study by Shacham M et al. revealed that a relatively low percentage of participants showed signs of dental anxiety [58]. The same low level of anxiety was also found in studies conducted on populations from Japan [59], Turkey [60], and Malaysia [61].

The IDAF scores indicate a moderate level of dental anxiety, with the mean score for all IDAF scores (IDAF—C, IDAF—S, IDAF—P) suggesting fairly high variability between participants but also a relatively constant level of phobias and anxiety related to dental treatments. The results obtained are consistent with the results of randomized clinical trials, which highlight reductions in dental anxiety after repeated dental interventions [61].

Done et al. found low levels of dental anxiety that may have been associated with scaling, orthodontic treatments, or implant–prosthetic rehabilitation, while mobile and mobilizable prosthetic treatments were associated with higher levels of dental anxiety [41].

Murillo-Benitez et al. demonstrated in their study that some therapeutic procedures can increase anxiety levels, including both the procedure itself and subjective factors such as the duration of treatment, the level of pain experienced [62], and the lack of an explanation and understanding of the treatment plan [63].

Saatchi et al. reported increased dental anxiety despite technological advances in modern dentistry [51,64].

Anxiety caused by the high prevalence of dental pain is often correlated with a low quality of life index [65,66,67]. A fear of infection or spontaneous bleeding, embarrassment due to the appearance of teeth, or pain accompanying various dental procedures are considered causes of dental anxiety by Bothaina et al. [64]. A fear of anesthetic injections is also associated with dental anxiety, and all of these contribute to affecting quality of life [68].

This study also showed that a fear of the dentist mainly influences the physical and social relationships domains of quality of life. The scores in the physical health domain were close in value to the scores in the social relationships domain. In contrast, the scores for the environmental domain were increased. Therefore, these domains influenced the quality of life of the subjects participating in the study.

Critical analysis revealed that certain domains, such as social relationships, did not show a significant association with anxiety across studies. One possible reason could be the complexity and diversity of interpersonal relationships, which can influence how an individual perceives and responds to stress. Some studies suggest that positive social relationships may serve as protective factors against anxiety, while negative relationships or interpersonal conflicts may contribute to the development or intensification of anxiety symptoms [69]. However, the link between social relationships and anxiety is not always uniform, as other factors, such as the perceived social support, relationship quality, and attachment style, can modulate this effect. Individual characteristics, such as personality traits, may also influence how social relationships affect anxiety. For example, individuals with an anxious attachment style may misinterpret social cues, which may contribute to increased anxiety, even in the context of good social relationships [70]. These variables may explain why some studies have not found a significant association between social relationships and anxiety, highlighting the need for a more nuanced model that includes multiple dimensions of social interactions and individual personalities.

In their study, Andre et al. highlighted the fact that social relationships and physical health are components that have a high impact on quality of life [71]. The results obtained by Zhang et al. demonstrated that psychological domains and social relationships are much more important [72].

Previous dental experiences and academic stress may play a significant role in how students perceive and experience anxiety. Recent studies have suggested that previous dental trauma or unpleasant experiences related to dental visits may contribute to the development of dental anxiety [73], and this may influence behaviors and attitudes towards dental treatments in the long term. Academic stress, a common factor among students, has also been associated with increased levels of anxiety, both in terms of exams and in terms of adapting to academic demands [74]. Studies suggest that the combination of academic stress and previous negative dental experiences may lead to heightened anxiety, especially in situations involving dental care. For example, some students who have had painful experiences at the dentist or who have not had access to adequate dental care may develop a persistent fear of dental visits, and this phenomenon may be exacerbated by academic pressures [75]. Thus, an integrated understanding of these factors could help identify the most vulnerable individuals and develop appropriate interventions to reduce dental anxiety.

Bothaina H. H et al. have highlighted that an increased level of anxiety can be a predictor of a low quality of life [64].

This study had a descriptive cross-sectional design, using validated methods for measuring dental anxiety (DAS-R CORAH, IDAF) and quality of life (WHOQOL-BREF), which provided a solid basis for interpreting the results. The group of 180 participants, with a balanced distribution of genders and socio-economic levels, allowed for a relevant comparative analysis. It evaluated multiple variables, including participants’ gender, socio-economic status, and time since their last visit to the dentist, which allowed us to build a complex picture of dental anxiety and its impact on quality of life.

This study also allowed for the identification of significant correlations between dental anxiety and the physical and psychological domains of quality of life, contributing to the understanding of the emotional impact of dental anxiety.

However, the cross-sectional design only allowed for an association between variables to be found, without us being able to demonstrate causal relationships. The study did not explore in depth the psycho-behavioral factors that may influence the relationship between dental anxiety and quality of life. Although it analyzed how an individual’s gender and socio-economic level influence dental anxiety, no statistically significant differences were identified, which may limit the overall conclusions of the study.

All of these determine the limitations of this study. In addition, the geographically limited sample (the study was conducted within the Faculty of Dental Medicine, University of Iași) may limit the generalization of the results to more diverse populations from other regions or countries. Also, the data collected through questionnaires were based on self-reporting, which may have introduced a response bias (e.g., the underestimation or overestimation of dental anxiety). The study did not take into account other psychological variables that could influence dental anxiety, such as the history of negative dental experiences or the coping strategies used by the participants. The use of a homogeneous sample (dental students from a single institution) significantly limits the external validity of the results. Also, the absence of qualitative data or patients’ perspectives on their anxiety represents a missed opportunity to deepen our understanding of the phenomenon, and last but not least, the absence of a longitudinal analysis did not allow us to determine the impact of dental anxiety on the quality of life in the long term.

## 5. Conclusions

This study highlights that dental anxiety is a common problem among dental students, with the majority of participants experiencing moderate levels of anxiety. Although anxiety did not show significant associations with demographic factors such as participants’ gender or socio-economic status, it was strongly linked to their perceived physical and psychological well-being. Dental anxiety had a significant impact on the physical and psychological dimensions of quality of life, with significant correlations being identified between dental anxiety and these aspects. However, no significant relationship was observed between dental anxiety and social relationships or the environmental domain, suggesting that anxiety does not influence interpersonal or environmental factors in the same way. This suggests that managing sensory experiences during dental visits could help improve a patient’s perception of their physical condition. The working hypothesis that there are correlations between dental fear and anxiety and dimensions of quality of life was verified.

Future research should consider longitudinal designs and larger, more diverse samples to explore the long-term effects of dental anxiety on quality of life. Qualitative data from patients could also provide a deeper understanding of the emotional and psychological factors that contribute to dental anxiety.

## Figures and Tables

**Table 1:** WHOQOL-BREF indicator domains [47].

	Equations for Computing Domain Scores	Raw Score	Transformed Score	
			4–20	0–100
Domain 1 Physical health	(6-Q3) + (6-Q4) + Q10 + Q15 + Q16 + Q17 + Q18	=		
Domain 2 Psychological	Q5 + Q6 + Q7 + Q11 + Q19 + (6-Q26)	=		
Domain 3 Social relationships	Q20 + Q21 + Q22	=		
Domain 4 Environment	Q8 + Q9 + Q12 + Q13 + Q14 + Q23 + Q24 + Q25	=		

**Table 2:** Demographic characteristics of the study group.

Variable		No.	%
Age group	Aged 19–29	120	66.7
	Aged 30–39	60	33.3
Gender	Male	88	48.9
	Female	92	51.1
Residence	Rural	69	38.3
	Urban	111	61.7
Socio-economic level	High level	88	48.9
	Medium level	85	47.2
	Low level	7	3.9
Time since last check-up	<1 year	109	60.6
	>1 year	71	39.4

**Table 3:** Distribution of anxiety levels by gender and socio-economic level.

			DAS-R CORAH				*p*	*r*	*F*
			No Anxiety	Moderate Anxiety	High Anxiety	Severe Anxiety			
DAS-R Total Score			59.4%	26.1%	8.3%	6.1%			
Gender	Female	Count	61	29	9	8	0.760	0.072	0.384
		%	57.0%	61.7%	60.0%	72.7%			
	Male	Count	46	18	6	3			
		%	43.0%	38.3%	40.0%	27.3%			
Socio-economic level	High level	Count	54	22	8	4	0.800	0.061	0.632
		%	50.5%	46.8%	53.3%	36.4%			
	Medium level	Count	50	22	7	6			
		%	46.7%	46.8%	46.7%	54.5%			
	Low level	Count	3	3	0	1			
		%	2.8%	6.4%	0.0%	9.1%			
Time since last check-up	<1 year	Count	71	21	9	8	0.067	0.047	0.468
		%	66.4%	44.7%	60.0%	72.7%			
	>1 year	Count	36	26	6	3			
		%	33.6%	55.3%	40.0%	27.3%			

Anova, *p* = 0.005.

**Table 4:** The mean values and standard deviations for variables assessed by the IDAF and other components related to fear and discomfort indicate the general trends in the participants.

IDAF Components	Mean	SD
IDAF—C (Core Module)	1.9050	1.07950
IDAF—P (Phobia Module—Phobia Component)	1.7444	0.43739
IDAF—P (Phobia Module—Anxiety Component)	1.8833	0.32192
IDAF—S (Stimuli Module)	2.5261	0.86266
Pain	2.7444	1.12425
Embarrassed/ashamed	2.1222	1.23567
Control	2.2333	1.09901
Sick/disgusted	2.7056	1.33996
Numbness	2.0778	1.27483
Not knowing	2.1889	1.27191
Treatment cost	3.2333	1.25560
Injection	2.3778	1.35842
Gagging/choking	2.8944	1.42796
Unsympathetic dentist	2.6833	1.30951

**Table 5:** Domain scores from WHOQOL-BREF.

Domain	Raw Score	Normalized Scores
Physical Health	12.34	44.04
Psychological	17.26	63.95
Social Relationships	12.44	41.25
Environment	32.36	49.62
Total Score = 74.4		

**Table 6:** Analysis of correlations between WHOQOL-BREF and DAS-R CORAH and IDAF scores highlighted significant relationships between dental anxiety and certain dimensions of quality of life.

WHOQOL-BREF Domains		DAS-R CORAH	IDAF			
			IDAF—C Core Module	IDAF—P Phobia Module	IDAF—P Anxiety Module	IDAF—S Stimulus Module
Physical domain	Pearson correlation	−0.136	−0.136	0.070	0.184 *	−0.129
	Sig. (2-tailed)	0.068	0.068	0.353	0.014	0.084
	N	180	180	180	180	180
Psychological domain	Pearson correlation	0.198 **	0.198	−0.056	−0.241 **	0.221 **
	Sig. (2-tailed)	0.008	0.008	0.456	0.001	0.003
	N	180	180	180	180	180
Social relations domain	Pearson correlation	−0.135	−0.135	0.084	0.028	−0.119
	Sig. (2-tailed)	0.071	0.071	0.263	0.712	0.111
	N	180	180	180	180	180
Environmental domain	Pearson correlation	0.009	0.009	0.011	0.198 **	−0.040
	Sig. (2-tailed)	0.903	0.903	0.888	0.008	0.597
	N	180	180	180	180	180

* The correlation is significant at the 0.05 level (2-tailed). ** The correlation is significant at the 0.01 level (2-tailed). IDAF-C (Core Module), IDAF-P (phobia), IDAF-P (anxiety), IDAF-S (Stimulus Module).

## Data Availability

The data supporting the reported results can be obtained upon request in the form of datasets available in the Community Dentistry Discipline, Department of Surgery, Faculty of Dental Medicine, “Grigore T. Popa” University of Medicine and Pharmacy, Iasi, Romania.
